# Leaf manganese concentrations reveal phosphorus-mining strategies and trait diversification of Myrtaceae in south-eastern Australia

**DOI:** 10.1093/aob/mcaf129

**Published:** 2025-06-17

**Authors:** Li Yan, Patrick E Hayes, Francis J Nge, Erin I E Rogers, Ian J Wright, Kosala Ranathunge, David S Ellsworth, Hans Lambers

**Affiliations:** School of Biological Sciences, The University of Western Australia, 35 Stirling Highway, Perth, WA 6009, Australia; School of Biological Sciences, The University of Western Australia, 35 Stirling Highway, Perth, WA 6009, Australia; National Herbarium of New South Wales, Botanic Gardens of Sydney, Locked Bag 6002, Mount Annan, NSW 2567, Australia; Hawkesbury Institute for the Environment, Western Sydney University, Locked Bag 1797, Penrith, NSW 2751, Australia; Hawkesbury Institute for the Environment, Western Sydney University, Locked Bag 1797, Penrith, NSW 2751, Australia; School of Natural Sciences, Macquarie University, Sydney, NSW 2109, Australia; School of Biological Sciences, The University of Western Australia, 35 Stirling Highway, Perth, WA 6009, Australia; Hawkesbury Institute for the Environment, Western Sydney University, Locked Bag 1797, Penrith, NSW 2751, Australia; School of Biological Sciences, The University of Western Australia, 35 Stirling Highway, Perth, WA 6009, Australia

**Keywords:** Eucalypts, leaf manganese, mycorrhiza, phosphorus acquisition, phylogeny, root carboxylates, species distribution

## Abstract

**Background and Aims:**

Phosphorus (P)-impoverished soils shape plant adaptation in biodiverse ecosystems worldwide, from Australian heathlands to Amazonian rainforests to southern China's karst regions. While non-mycorrhizal lineages like Proteaceae and Cyperaceae use carboxylate exudation that mobilise P, and are celebrated for such strategies, the mechanisms allowing mycorrhizal Myrtaceae—especially eucalypts—to thrive in these soils without fungal assistance remain unclear. Given Myrtaceae's dominance in P-impoverished Australian ecosystems, a key question arises: How do mycorrhizal plants succeed in P-impoverished environments without relying on fungal symbiosis? We challenge the paradigm that carboxylate-driven P acquisition is exclusive to non-mycorrhizal species.

**Methods:**

Using leaf manganese concentrations ([Mn]) as a proxy for carboxylate exudation, we assessed trait diversification across Myrtaceae genera. We collected leaf and soil samples from 34 species of eucalypt (*Angophora*, *Blakella*, *Corymbia*, *Eucalyptus*) and other Myrtaceae from 18 sites in south-eastern Australia.

**Key Results:**

Our findings reveal consistently high leaf [Mn] in many Myrtaceae, comparable to that in known carboxylate-releasing species, indicating intensive P mining. This suggests convergent evolution of carboxylate exudation in mycorrhizal Myrtaceae, fundamentally reshaping our understanding of nutrient acquisition in symbiotic plants. Significant interspecific variation was observed, with *Angophora* showing markedly higher [Mn] than *Eucalyptus*, suggesting divergent P-acquisition strategies within Myrtaceae. Weak phylogenetic signals for leaf [Mn] and [P] in eucalypts imply repeated evolutionary change in these traits, similar to what is known in other Australian species adapted to P scarcity.

**Conclusions:**

By demonstrating carboxylate-driven P mining in mycorrhizal Myrtaceae, we redefine the mechanisms behind their dominance in low-P environments. Trait diversity—linked to variation in carboxylate-mediated P acquisition and plant-soil feedbacks—likely drives niche differentiation and genus-level distribution across south-eastern Australia. Connecting leaf [Mn] to carboxylate-driven P mining advances our understanding of trait evolution in Myrtaceae and provides a framework for predicting plant-soil interactions in P-impoverished ecosystems globally.

## INTRODUCTION

Australia is well known for its phosphorus (P)-impoverished soils (Beadle, 1953, McArthur, 1991), which reflect the nature of the parent material and the age of the landscapes (Viscarra Rossel and Bui, 2016, Kooyman *et al*., 2017). Soil P availability in most of Australia is much lower than the global average (Batjes, 2011, Jiang *et al*., 2024), and it has had a profound influence on the Australian flora (Beadle, 1966, Hayes *et al*., 2021), with the greatest plant diversity being associated with the most severely P-impoverished soils (Adam, 2012, Lambers *et al*., 2014). Numerous adaptations have evolved in the Australian flora to cope with the low P status of the soils. These include mechanisms to efficiently acquire P from severely P-impoverished soils, e.g. cluster roots in most Proteaceae, Casuarinaceae, and many Fabaceae (Shane and Lambers, 2005*a*, Nge *et al*., 2020), dauciform roots in many Cyperaceae (Lamont, 1974, Shane *et al*., 2006), capillaroid roots in Restionaceae (Lamont, 1982, Lambers *et al*., 2014), and sand-binding roots in Haemodoraceae (Smith *et al*., 2011). Most of the species expressing these morphological adaptations are non-mycorrhizal and exhibit carboxylate-releasing P-acquisition strategies for mining soil P (Lambers, 2022). In addition, once P is captured, many of these species exhibit strategies to efficiently use P and also to extend the residence time of nutrients in plant organs before being lost as litter, e.g. long leaf lifespans, and highly efficient remobilisation of P from senescing leaves (Wright and Westoby, 2003, Denton *et al*., 2007, Lambers *et al*., 2012, Hayes *et al*., 2014, Sulpice *et al*., 2014, Guilherme Pereira *et al*., 2019).

Carboxylate-releasing P-mining strategies can be proxied by analysing leaf manganese (Mn) concentrations ([Mn]), because carboxylates in combination with protons not only mobilise soil P but also a range of micronutrients, including Mn (Lambers *et al*., 2015, Pang *et al*., 2018). Manganese uptake in plants involves a transport system that takes up iron (Fe) as well as a range of other elements and is poorly controlled in roots (Baxter *et al*., 2008, Liu *et al*., 2024). Whenever Mn availability in the rhizosphere is enhanced by the release of carboxylates and/or protons, leaf [Mn] increases (Lambers *et al*., 2015, Pang *et al*., 2018, Etabo *et al*., 2024). Conversely, mycorrhizal hyphae intercept Mn, and hence plants with effective mycorrhizas typically have a low leaf [Mn] (Nogueira *et al*., 2004, Canton *et al*., 2016, Garcia *et al*., 2024). Since leaf [Mn] strongly depends on edaphic and climatic conditions (Lambers *et al*., 2021), there is a need to include reference species to indicate carboxylate release, particularly when considering diverse locations (Zhou *et al*., 2020, Michelini *et al*., 2025, Tang *et al*., 2025, Yan *et al*., 2025). A suitable positive reference is a Proteaceae that is known to release large amounts of carboxylates (Shane and Lambers, 2005*a*). Proteaceae tend to exhibit high leaf [Mn], but there are exceptions, e.g. *Xylomelum* species, which do not produce functional cluster roots (Zhong *et al*., 2021) and some *Hakea* species, which release carboxylates without protons (Roelofs *et al*., 2001) but with cations such as K^+^ and Ca^2+^ (Gille, 2024). Xanthorrhoeaceae and Zamiaceae exhibit low leaf [Mn] and are suitable as negative references which indicate no significant carboxylate release (Zhong *et al*., 2021, Lambers *et al*., 2022). When tested in nutrient solution in a glasshouse, a commonly used negative reference species, *Xanthorrhoea preissii*, does not release carboxylates (Wang *et al*., 2025). When no suitable reference species are available, young leaves can be used, as Mn only accumulates in older leaves, increasing with leaf age (Shane and Lambers, 2005*b*, Lambers *et al*., 2021). Unlike Proteaceae and Cyperaceae, none of the dominant and widespread eucalypts have specialised carboxylate-releasing structures, but some release carboxylates from their entire root system (Zhou *et al*., 2022), possibly as a mechanism to release surplus carbon (Prescott *et al*., 2020, Prescott, 2022).

Eucalypts comprise about 840 species belonging to four genera, *Angophora*, *Blakella*, *Corymbia* and *Eucalyptus*, most of which are endemic to Australia; *Blakella* was until recently considered to belong to the genus *Corymbia* (Nicolle, 2015, Crisp *et al*., 2024, Cook *et al.*, 2025, Nicolle *et al*., 2025). A recent broad survey of plant species across Australia showed that many eucalypts exhibit high leaf [Mn], suggesting that these mycorrhizal plant species may rely on carboxylate release to acquire P (Lambers *et al*., 2021), and less on their associated mycorrhizal fungi, which would intercept Mn and lead to low leaf [Mn] (Bethlenfalvay and Franson, 1989, Kothari *et al*., 1991, Nogueira *et al*., 2007, Canton *et al*., 2016). Lambers *et al*. (2021) investigated carboxylate release in *E. patens* grown in low-P solutions in a glasshouse, showing that it does, indeed, release carboxylates, mainly oxalate as opposed to citrate and malate in Proteaceae, Cyperaceae and Restionaceae (Shane *et al*., 2004, Lambers *et al*., 2014). Zhou *et al*. (2022) showed that other eucalypts in Western Australia, including *E. diversicolor*, *E. marginata* and *C. calophylla* also exhibit high leaf [Mn] in the field and release carboxylates when grown in nutrient solution in a glasshouse. This led to the question: how common is high leaf [Mn] and inferred carboxylate release in Myrtaceae, especially in eucalypts? We hypothesised that Myrtaceae in P-impoverished soils use carboxylate exudation, as inferred by their high leaf [Mn], to complement mycorrhizal P acquisition, enabling their dominance in these ecosystems.

Carboxylate-releasing nutrient-acquisition strategies and high P-use efficiency may be linked to the diversification of this diverse and ecologically dominant plant group across Australia (Yan *et al*., 2019, Hayes *et al*., 2021, Lambers, 2022). Indeed, this link has been demonstrated for Proteaceae, where lower leaf [P] is correlated with faster diversification rates of species-rich genera (Hayes *et al*., 2021). Multiple independent transitions to higher P-use efficiency (low leaf [P]) have been demonstrated for most *Hakea* and *Banksia* (Proteaceae; Hayes *et al*., 2021), though all of them have low leaf [P] compared with global average values (Wright *et al*., 2004), suggesting this trait is evolutionarily labile in Proteaceae. Similarly, cluster roots, which are indicative of carboxylate-exudation strategies, are phylogenetically dispersed across *Daviesia* (Fabaceae) and three other closely related genera (*Gompholobium, Sphaerolobium,* and *Viminaria*; Nge *et al*., 2020). It is currently unknown whether these traits are evolutionary conserved or labile across eucalypts (*i.e.* whether they are constrained to selected clades or widespread throughout the group). Determining the extent of these traits for eucalypts is important for delineating which species or eucalypt groups gain dominance on low-nutrient soils and gain insight into how they do so. This is important based on the ecological importance of eucalypts in Australia as well as the importance of this genus in plantations in other parts of the world.

Our main aim was to survey a wide range of eucalypts (*Angophora*, *Blakella*, *Corymbia*, *Eucalyptus*) in south-eastern Australia, as the south-eastern and Western Australian floras have been largely isolated for the last ∼15 million years since the uplift of the Nullarbor Plain (Crisp and Cook, 2013). This allowed us to explore, using leaf [Mn] as a proxy, how common the carboxylate-releasing nutrient-acquisition strategy is, and to investigate if it is linked to the diversification and evolution of eucalypts. We therefore sampled species from different locations with different soil characteristics. At each location, we also sampled positive and negative reference species as well as topsoil for chemical analyses.

## MATERIALS AND METHODS

### Study sites

**Fig. 1.**
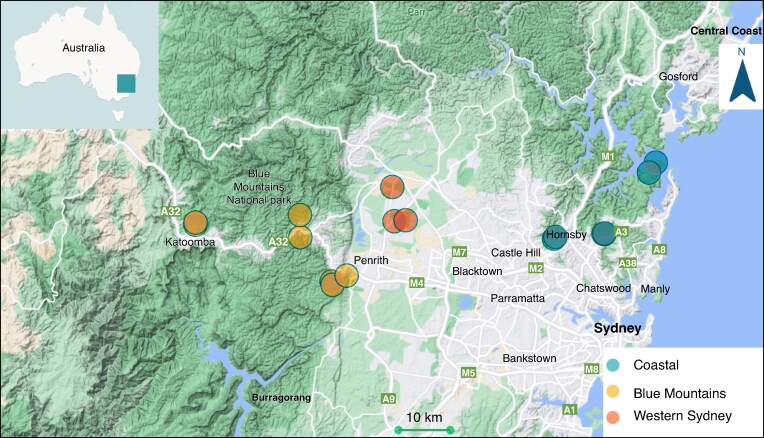
Study sites in New South Wales, Australia. The map was created through the CARTO Builder.

A total of 18 sites from three regions were selected in New South Wales, Australia (33°37′-33°47′S, 150°19′-151°17′E). Sites were selected based on the distribution of various Myrtaceae, particularly eucalypts, *i.e. Angophora*, *Blakella*, *Corymbia* and *Eucalyptus* (Fig. 1). These sites included seven from the Blue Mountains region (Davies Park, Grose Road, Euroka, Lapstone and Minnehaha Reserve), eight from a coastal region (four sites in Kuring-Gai Chase National Park, two in Kuring-Gai Wildflower Garden as examples of pristine, remnant natural vegetation, and two in Dog Pound Creek Reserve), and three from Western Sydney (two in Castlereagh Reserve and one at EucFACE). Soils at all sites were considered to be low in available soil P (Supplementary Data Tables S1 and S2).

### Leaf and soil collection

Leaf and soil samples were collected from all 18 sites in November 2022. In total, we collected a diverse range of Myrtaceae, including 30 eucalypt species (six *Angophora*, one *Blakella*, one *Corymbia*, and 22 *Eucalyptus* species), as well as two *Melaleuca* and two *Gaudium* species (known as *Leptospermum* before revision of the genus; Wilson and Heslewood, 2023). *Banksia* species were chosen as positive reference species when present, as they produce cluster roots and exude carboxylates under low-P conditions (Shane and Lambers, 2005*a*). However, due to their absence at two sites (Kuring-Gai Chase National Park 1 and Lapstone), they were substituted by *Persoonia*, which belongs to the same family; it lacks cluster roots but does release root carboxylates (Lambers *et al*., 2021). *Xanthorrhoea* species served as negative reference species at most sites, based on published results, suggesting very low/no release of root carboxylates (Zhong *et al*., 2021, Zhou *et al*., 2022, Wang *et al*., 2025). *Acacia* and *Macrozamia* species, and young leaves of *Eucalyptus* species (at the Lapstone site) were selected as negative references when *Xanthorrhoea* was absent. In cases where reliable positive or negative reference species were unavailable, average values obtained from all other sites were used instead. Average positive references (APR) were used at Western Sydney-EucFACE and Coastal-Dog Pound Creek Reserve 2; average negative references (ANR) were used at Blue Mountains-Gross Road, Blue Mountains-Minnehaha 1, Western Sydney-Castlereagh Reserve 2 and Coastal-Kuring-Gai Wildflower Garden 2. At each site, four to five mature healthy individuals of similar height were selected for each species and five to 10 g of leaf material was sampled from each plant. The youngest fully mature intact leaves were selected.

Soil samples were collected from five locations at each site, with locations centrally located within the distribution of sampled plants. Within each location, leaf litter was removed and three soil cores (10 cm depth) were taken and bulked (200 to 300 g).

### Analyses of leaf and soil samples

After collection, all plant samples were dried at 70°C for >72 h and ground to a fine powder using a ceramic ball mill (Mixer Mill MM 400, Retsch, Haan, Germany). Nitrogen (N) concentrations were analysed using a LECO Trumac Analyser (LECO model CHN828, St. Joseph, MI, USA). Other elements (P, potassium (K), calcium (Ca), copper (Cu), zinc (Zn), Mn, Fe and magnesium (Mg)) were analysed via inductively coupled plasma-mass spectrometry (ICP-MS, Perkin Elmer ICPMS NexION 2000B with ESIFAST autosampler, Waltham, Massachussetts, US) and Inductively Coupled Plasma Optical Emission Spectrometer (ICPOES, Perkin Elmer Avio 500, Waltham, Massachussetts, US) after nitric acid digestion, following the American Public Health Association (APHA) guidelines for metal analysis (APHA, 2018).

Soil samples were sieved to <2 mm, dried at 40°C, and stored dry before analysis. The total concentrations of Ca, Mg, K, P, Zn, Mn, Fe and Cu in the soil were measured through aqua regia block digestion (Hotblock 200- Environmental Express Inc. Charleston USW Model SC2050-54V, Charleston, South Carolina, US) and determination by ICP-MS and ICPOES (Rayment and Lyons, 2011). Soil pH was determined in both water (1:5; soil: water) and 0.01 M CaCl_2_ (1:5; soil: CaCl_2_; with stirring during measurement). Soil electrical conductivity (EC) was assessed (1:5; soil : water). Plant-available soil P concentration was analysed using the Olsen P method (Olsen, 1954) as well as extraction in deionised water (1:5; soil: water; 30 min), with P concentrations measured using colorimetric flow injection analysis (Quik-Chem 8000, Lachat Instrument, Milwaukee, Wisconsin, US). All plant and soil samples were analysed at Southern Cross University's Environmental Analysis Laboratory (www.scu.edu.au/eal).

### Data analyses

Since our primary interest was to compare leaf [Mn] of the target species with the reference species (Table 1) rather than among all species, Welch *t*-tests were identified as the most appropriate statistical test (Stewartoaten *et al*., 1992, Ruxton, 2006). In addition to comparing the absolute leaf [Mn] between target Myrtaceae and reference species, we also computed normalised leaf [Mn] values by considering both negative and positive references at each site. In one way, we presented the difference between target species and the negative reference at each site, the value of a target species was subtracted from the mean of the negative reference (Supplementary Data Fig. S3), while the Welch *t*-test was also used to compare this difference with the absolute leaf [Mn] (Fig. 3). Similarly, we presented the difference between targeted species and the positive reference at each site, the value of a targeted species being subtracted from the mean of the positive reference (Supplementary Data Fig. S4), while the Welch *t*-tests was used again but with the absolute leaf [Mn] (Fig. 3). Additionally, we compared leaf [Mn] of targeted species across different sites by standardising the absolute leaf [Mn] using the leaf [Mn] of respective positive and negative references at each site using Equation 1 (Supplementary Data Fig. S5; Tang *et al*., 2025):


(1) $$\begin{array}{rl} & \mathrm{Relative}\;\mathrm{leaf}\;[\mathrm{Mn}](\text{\%})=\frac{\mathrm{Target}\;[\mathrm{Mn}]-\;\mathrm{Neg}\;\mathrm{ref}\;[\mathrm{Mn}]}{\mathrm{Pos}\;\mathrm{ref}\;[\mathrm{Mn}]-\;\mathrm{Neg}\;\mathrm{ref}\;[\mathrm{Mn}]}\;\times 100 \end{array}$$

**Table 1.** Criteria for root carboxylate release proxied by leaf manganese concentration ([Mn]).

Criteria	Outcome	Data
A significantly higher leaf [Mn] than the negative reference	The target species **does** exude root carboxylates (or depends on facilitation of its nutrient acquisition by its neighbours, Shen *et al.*, 2024*b*; Staudinger *et al*., 2024)	Fig. 3 and Supplementary Data Figs S3 and S4
No significant difference compared with the negative reference	The target species **does not** exude root carboxylates	Fig. 3 and Supplementary Data Figs S3 and S4
A similar or significantly higher leaf [Mn] than the positive reference	The target species has a **strong capacity** to exude root carboxylates	Fig. 4 and Supplementary Data Figs S3 and S4

Bold text indicates different root carboxylate exudation types.

To compare the variation in leaf nutrient concentrations among sites, appropriate generalised least square models (GLS) were used to examine correlations between the response variables (leaf [N], leaf [P], leaf [Mn] and leaf N:P ratio) and the predictor variable (species or site). The models (Supplementary Data Table S3) were developed incrementally, each incorporating different weight structures to address potential heteroscedasticity in the data. The final models were selected based on the lowest Akaike's Information Criterion corrected (AICc) values (Bartoń, 2024) by using the R package ‘nlme’ (Pinherio *et al.*, 2017), and *post hoc* comparisons using Tukey's honestly significant difference test (*P* < 0.05) (Pinheiro and Bates, 2000).

Principal component analysis (PCA) and Pearson correlation analyses were conducted on the data using the packages ‘FactoMineR’ (Le *et al*., 2008) and ‘stats’ in R (2024). Kaiser-Meyer-Olkin (KMO) measure (>0.6) and Bartlett's test of sphericity (*P* < 0.05) were inspected for the suitability of PCA. Log transformation was conducted for Pearson correlations. All statistical analyses were done and figures were produced in R Version 4.4.1 (R Core Team, 2024) and packages ‘ggplot2’ (Wickham, 2016), ‘cowplot’ (Wilke *et al*., 2019), ‘corrplot’ (Wei *et al*., 2017), ‘factoextra’ (Kassambara and Mundt, 2020).

### Phylogenetic signal and diversification analyses

We obtained a published eucalypt phylogeny from Thornhill *et al*. (2019) which included 27 of the 30 sampled eucalypt species in our study. We conducted phylogenetic analyses based on these 27 taxa that are present in the phylogeny and excluded the other three—*Angophora crassifolia, Eucalyptus punctata* (subg. *Symphomyrtus*) and *Eucalyptus signata* (subg. *Eucalyptus*; following our collection trip, we noticed a revision of this species and *Eucalyptus signata* and *E. racemosa* have been merged). To test if leaf [P] and [Mn] are evolutionary labile across eucalypts, we assessed for the strength of the phylogenetic signal of these traits. Both traits were log-transformed prior to all analyses below. The phylogeny was also pruned to the 27 as phylogenetic signal analyses require complete datasets with no missing values. Blomberg's K was calculated for these traits using the *phylosig* function in the package ‘phytools’ Version 1.0.3 (Revell, 2012) in R Version 3.5.0 (R Core Team, 2024). We calculated the K value based on 10 000 replicates to assess for statistical significance (*P* < 0.05). Both traits were also visualised and mapped onto the phylogeny using the *contMap* function in ‘phytools’ in R.

To test whether leaf [P] and leaf [Mn] are correlated with diversification across eucalypts, we first conducted a Bayesian analysis of macroevolutionary mixtures (BAMM; Rabosky, 2014), using the full dated phylogeny of Thornhill *et al*. (2019) to obtain speciation tip rates (the rate of new species formation at terminal branches of the phylogeny) for each species. Using the wider phylogeny instead of the pruned 27-tip subset would allow for more accurate inference of diversification rates as rates depend on the divergence of sister taxa and the wider clades that were not sampled in this study. For BAMM, we specified a conservative global sampling fraction of 70 % for the phylogeny to account for undescribed species or unsampled taxa in Thornhill *et al*. (2019). From the BAMM analysis, we extracted the tip speciation rates for each of the 27 sampled species in this study using the *getTipRates* function in the R package ‘BAMMtools’ Version 2.1.10 (Rabosky *et al*., 2014). We used BAMM to obtain tip rates instead of model-free methods such as the DR statistic (species-level lineage diversification rate) as BAMM is more accurate than DR (Title and Rabosky, 2019). We also focused on speciation instead of net diversification rates (speciation—extinction) as estimating extinction rates from extant phylogenies is fraught with difficulties (Rabosky, 2010, Wiens, 2024). Speciation tip rates were log-transformed prior to correlation analyses. We conducted Spearman rank correlation tests for tip rates and leaf [P], leaf [Mn] in R. To account for phylogenetic relatedness, we also conducted phylogenetic generalised least square regression (PGLS) for these traits using the *gls* function in the R package ‘nlme’ Version 3.1.145 (Pinherio *et al.*, 2017).

## RESULTS

### Soil characteristics

Soils at all sites were acidic, with the soil pH (CaCl_2_) of all 18 sites below 5.0 (Supplementary Data Table S1). The site with the lowest soil pH was Coastal-Dog Pound Creek Reserve 2 with a pH of 3.4. We observed the highest soil pH (4.9) at Coastal-Kuring-Gai Chase NP1. Most sites exhibited low soil total [P], with half of them showing soil [P] below 50 mg P kg^−1^. Coastal-Kuring-Gai Chase NP1 stood out as having the highest soil total [P] at 313 mg P kg^−1^. All sites displayed relatively low extractable soil P concentrations (Olsen [P]) of around 2 mg P kg^−1^, except for Coastal-Dog Pound Creek Reserve 2, which had the highest Olsen [P] of 9.1 mg P kg^−1^. Although there were slight differences in soil [Mn] among sites, most values were below 25 mg Mn kg^−1^; the lowest soil [Mn] was at Kuring-Gai Chase NP2 and at Dog Pound Creek Reserve 2 in a coastal region, where soil [Mn] was only 2.2 mg Mn kg^−1^, whereas Kuring-Gai Chase NP1 exhibited the highest soil [Mn] at 109 mg Mn kg^−1^. Interestingly, this particular site also demonstrated the highest concentrations of Fe, Mg, Ca, Cu and Zn (Supplementary Data Tables S1).

### Leaf nitrogen and phosphorus concentrations

**Fig. 2.**
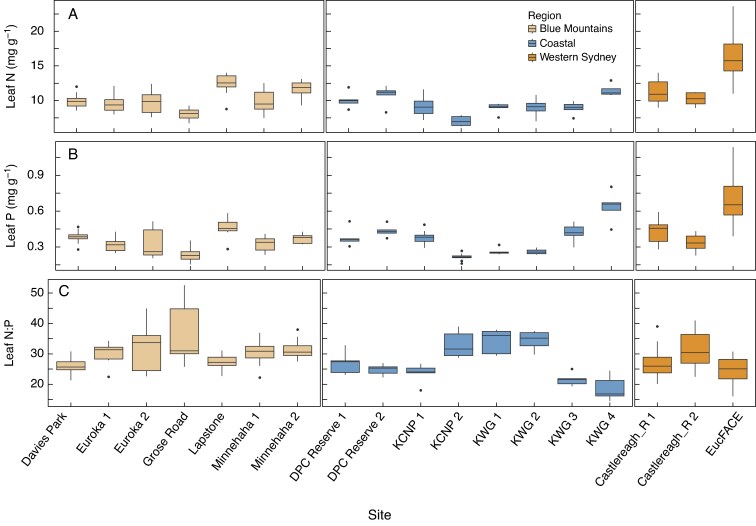
Leaf nitrogen (N, A) and phosphorus (P, B) concentrations, and leaf N:P ratio (C) for the target Myrtaceae species across all 18 sites. This analysis excluded reference species; *n* = 193 individuals across 34 species. Each boxplot includes contributions from both within-species and among-species trait variation. In this figure, we primarily aim to illustrate the overarching trends in leaf [N], [P], and N:P ratios across diverse sites, rather than conducting direct statistical comparisons among sites. This approach avoids potential misinterpretations arising from the heterogeneity in soil conditions (e.g. pH, nutrient availability) among locations which might confound comparisons among sites. Statistical analyses of leaf [N], [P], and N:P ratios are shown in Supplementary Data Fig. S1.

The EucFACE site in the Western Sydney region exhibited the highest mean concentrations of both leaf N and P (Fig. 2A, B; Supplementary Data Fig. S1) with an average of 16.2 mg N g^−1^ and 0.7 mg P g^−1^ for the four Myrtaceae species sampled. By contrast, the lowest leaf [N] and [P] were 8.1 mg N g^−1^ and 0.3 mg P g^−1^ for species found at Kuring-Gai Chase NP2 in a coastal region. The average leaf [N] and [P] across all 18 sites were 10.6 mg N g^−1^ and 0.4 mg P g^−1^, respectively. Among all 18 sites, there were 12 sites that had a leaf [P] lower than the average of 0.4 mg P g^−1^.

There were five species that had a leaf [P] greater than 0.6 mg P g^−1^, and four of them (*A. floribunda*, *A. subvelutina*, *E. tereticornis* and *M. decora*) were from the EucFACE site (Supplementary Data Fig. S2). The highest leaf [P] was 0.9 mg P g^−1^ for *E. tereticornis* at Western Sydney-EucFACE which also had the highest leaf [N] of 19.1 mg N g^−1^. The lowest leaf [P] was 0.18 mg P g^−1^ for *G. trinervium* at Grose Road in the Blue Mountain region, followed by *A. hispida* (0.23 mg P g^−1^) and *E. haemostoma* (0.21 mg P g^−1^) at Ku-ring-gai Chase National Park 2 in a coastal region (Supplementary Data Fig. S2).

The mean leaf N:P ratio across all 18 sites was 28, with 17 sites above 21 (Fig. 2C); values > 20 indicate that plant growth is limited by P, according to Güsewell (2004). The site of Ku-ring-gai Wildflower Garden 4 in a coastal region had a leaf N:P ratio of 19. Half of the sites displayed a leaf N:P ratio >30, while the highest value of 36 was observed at Blue Mountain-Grose Road. The highest leaf N:P ratio was 48 for *G. trinervium* from the site of Grose Road in the Blue Mountain region, while *A. crassifolia* had the lowest mean leaf N:P ratio of 19 at Ku-ring-gai Wildflower Garden 4 in a coastal region (Supplementary Data Fig. S2).

### Leaf manganese concentrations

**Fig. 3.**
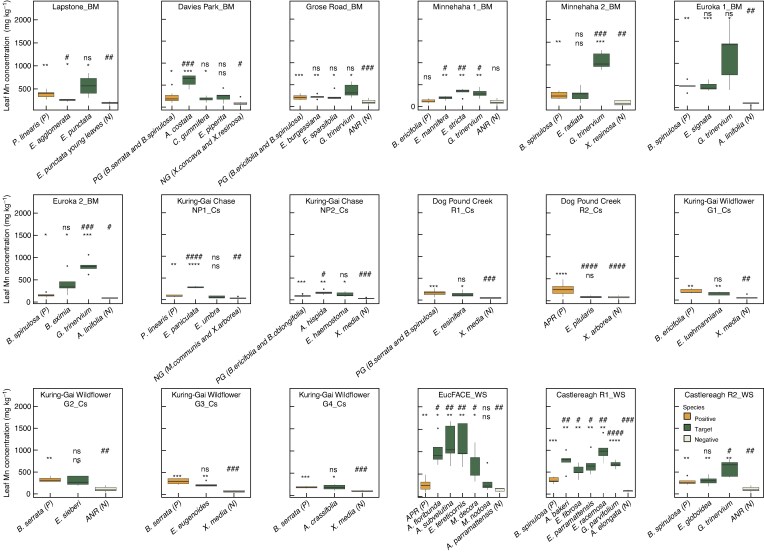
Leaf manganese (Mn) concentrations for target species (green), positive reference species (P, brown box at the very left) and negative reference species (N, grey box at the very right) from 18 sites (across three regions: BM, blue mountains; Cs, coastal; WS; western Sydney) in New South Wales, Australia. The Welch *t*-test was used to compare the target species and positive reference species at each site (#, *P* < 0.05; ##, *P* < 0.01; ###, *P* < 0.001; ####, *P* < 0.0001), and the target species and negative reference species at each site (*, *P* < 0.05; **, *P* < 0.01; ***, *P* < 0.001; ****, *P* < 0.0001); ns indicates no significant difference; *n* = 4–5 replicates per species. In addition, both the symbols on the top of P and N report the comparison of P vs N references. Where two negative or two positive reference species were sampled at a single site, they were grouped together, NG and PG, respectively. Where no suitable reference species were available the average positive reference (APR) and average negative reference (ANR) was used (see Materials and Methods for details).

All six *Angophora* species had significantly higher leaf [Mn] than those of their negative reference (Fig. 3 and Supplementary Data Fig. S3). Moreover, five *Angophora* species (*A. floribunda, A. subvelutina, A. costata, A. bakeri and A. hispida*) had much higher leaf [Mn] than the positive reference species (Fig. 3 and Supplementary Data Fig. S4), with *A. floribunda* and *A. subvelutina* having an average leaf [Mn] over 1000 mg Mn kg^−1^ (Fig. 3 and Supplementary Data Fig. S4). Both *Corymbia* and *Blakella* species (*C. gummifera* and *B. eximia*) had higher leaf [Mn] than negative reference species, but not higher than the positive reference species. Seventeen *Eucalyptus* species (77 %) out of 22 had significantly higher leaf [Mn] than the negative reference species. Moreover, seven of these 17 *Eucalyptus* species had higher leaf [Mn] than the positive reference samples (Fig. 3; see also Table 1 criteria). We found large variation in leaf [Mn] among *Eucalyptus* species; *E. tereticornis* from Western Sydney-EucFACE presented the highest leaf [Mn] of 1166 mg kg^−1^ of all *Eucalyptus* species (Fig. 3); *E. umbra* at Coastal-Kuring-Gai Chase NP1 had the lowest leaf [Mn] of 63 mg kg^−1^.

Both *Gaudium* species, present at seven of the sites, had higher leaf [Mn] than negative references at all sites. These values for *Gaudium* were much higher than those of the positive references at five of all seven sites. *Gaudium trinervium* at Blue Mountain-Euroka 1 presented the highest leaf [Mn] of all species among all sites (1200 mg Mn kg^−1^, Fig. 3).

### Principal component analysis of leaf nutrient concentrations and soil chemical properties

**Fig. 4.**
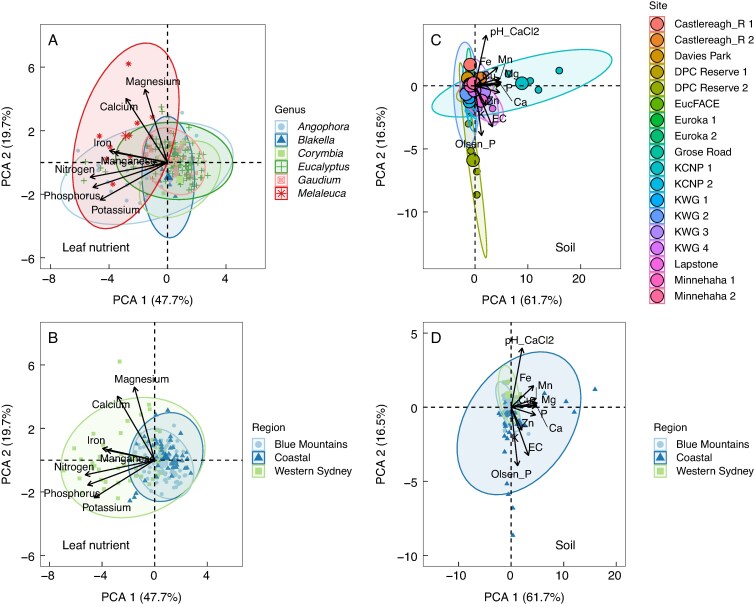
Principal component analysis biplot of leaf nutrient concentrations among genera (A) and regions (B) and soil chemical properties among sites (C) and regions (D). For leaf nutrient concentrations *n* = 193 individuals across 34 different species and *n* = 90 for soil chemical properties.

Principal component analysis of leaf nutrient concentrations showed that *Melaleuca* was grouped slightly separate from the other genera, tending to be grouped to the left along the horizontal principal component axis (PC1), and associated with higher concentrations of N and P (Fig. 4A). *Angophora* and *Eucalyptus* also varied along PC1 but overlapped more with the other Myrtaceae genera. Manganese and Fe concentrations were closely associated with each other and mainly varied along PC1, along with N, P and K. By contrast, Mg and Ca mainly varied along the vertical axes (PC2) and likely accounted for the greater vertical variation in *Melaleuca*. Plants within the Western Sydney region showed a larger variation in leaf nutrient concentrations than those in the other regions, while those in the Blue Mountains showed the least variation; however, all regions were overlapping (Fig. 4B).

Soil Mn, Ca, Cu, Mg and P concentrations were closely associated with one another and contributed strongly to PC1 in the soil PCA (Fig. 4C, D). Soil pH, Olsen [P] and EC contributed strongly to PC2, with soil pH inversely associated with Olsen [P] and EC. Soil chemical properties were most varied within the Coastal region, while the Blue Mountains and Western Sydney regions showed the least variation; however, all regions were strongly overlapping.

### Correlations among leaf and soil nutrient concentrations

Leaf [N] and [P] were strongly positively correlated (*r* = 0.85, *P* < 0.05) (Supplementary Data Fig. S6A), while soil pH and Olsen [P], soil pH and EC showed the strongest negative correlations among all soil parameters. Different genera of Myrtaceae showed distinct correlations among leaf nutrients (Supplementary Data Fig. S6B–D). *Angophora* showed the strongest correlations between leaf metals compared with *Eucalyptus* and *Gaudium*, especially between leaf [Mn] and [Fe] (*r* = 0.81, *P* < 0.05), followed by leaf [Ca] and [Cu] (*r* = 0.58, *P* < 0.05). We observed a strong correlation between leaf [N] and [Fe] (*r* = 0.84, *P* < 0.05) for *Angophora*. Different from *Angophora*, *Eucalyptus* showed weaker correlations between leaf [metals] (Supplementary Data Fig. S6C), but a stronger correlation between leaf [P] and [metals], for example, with leaf [Zn] (*r* = 0.7, *P* < 0.05) and leaf [Fe] (*r* = 0.64, *P* < 0.05). *Gaudium* did not have strong correlations compared with *Angophora* and *Eucalyptus*, except a moderately positive correlation between leaf [P] and [Cu] (*r* = 0.54, *P* < 0.05), leaf [N] and [Cu] (*r* = 0.48, *P* < 0.05).

### Phylogenetic signal and correlates of speciation rates

**Fig. 5.**
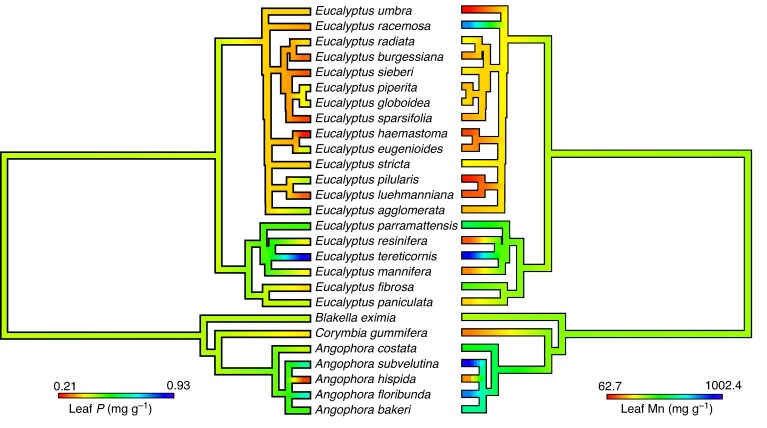
Maximum likelihood ancestral reconstructions of the concentrations of mature leaf phosphorus ([P]) and manganese ([Mn]) for eucalypts performed using the *contMap* function in phytools (Revell, 2012). We used the published phylogeny of Thornhill *et al*. (2019).

Some clades of eucalypts had lower leaf [P] than others (Fig. 5). In particular *Eucalyptus* subgenus *Eucalyptus sensu* (Crisp *et al*., 2024) (sampled *E. umbra–E. agglomerata* clade) had lower leaf [P] and [Mn] than *E.* subg. *Symphyomyrtus* (*E. parramattensis–E. paniculata* clade) and other genera of eucalypts. However, we detected only a weak phylogenetic signal for both leaf [P] and [Mn] across eucalypts based on Blomberg's K (Supplementary Data Table S4), with the traits having evolved more slowly than expected under Brownian motion. We detected a negative correlation between speciation tip rates and both leaf [P] and [Mn] based on Spearman rank analyses. However, only the leaf [P] correlation result was significant (*P* < 0.05; Supplementary Data Table S5). Our PGLS analyses indicate there was no significant correlation between leaf [P] or [Mn] and tip rates (Supplementary Data Table S6).

## DISCUSSION

Although Myrtaceae lack specialized root structures as found in Proteaceae and Cyperaceae, a similar effective release of root carboxylates appears to be widespread in this family, particularly in eucalypts, based on their common high leaf [Mn]. Furthermore, the present results reveal significant intergeneric differences in leaf [Mn] among Myrtaceae genera, for example, *Angophora* and *Eucalyptus*, suggesting diverse strategies for nutrient uptake and soil interactions and therefore plant distribution and related diversity. These findings are particularly important in the context of low soil P availability in south-eastern Australia, as leaf [Mn] is known to proxy rhizosphere carboxylates, which play a critical role in mobilising soil nutrients but remain largely unexplored in mycorrhizal fungi-associated plant species. However, a number of recent studies have shown high leaf [Mn] in a range of mycorrhizal species (Lambers *et al*., 2021, Zhou *et al*., 2022, 2024, Wang *et al*., 2025, Yan *et al*., 2025). Some of those species were grown in nutrient solution, confirming they release carboxylates (Lambers *et al*., 2021, Zhou *et al*., 2022, 2024, Wang *et al*., 2025).

Elevated CO_2_ was shown to cause more soil P to be locked up by soil microorganisms in mature *Eucalyptus* forests, thus restricting plant access to P (Jiang *et al*., 2024). Our analysis sheds light on how and which *Eucalyptus* forests liberate P, even if it is ultimately captured by other organisms. For example, *Eucalyptus urophylla* increases the release of organic acids with antibacterial effects (Wu and Yu 2019) which would further decrease P cycling in the plant-soil-microorganisms continuum. Investigating P-acquisition strategies is therefore important, both for P-impoverished natural ecosystems and for *Eucalyptus* plantations, especially in plantations that experience soil P limitation such as in southern China (Wu *et al*., 2014, Yao *et al*., 2023) and Brazil (Bassaco *et al*., 2018, Rocha *et al*., 2019).

### Soil characteristics and phosphorus limitation in south-eastern Australia

The acidic soils across the studied sites, with pH values below 5.0, indicate a challenging environment for nutrient availability, particularly P. The low soil total [P], with many sites showing less than 50 mg P kg^−1^, coupled with low Olsen [P], suggest that P is likely a primary limiting nutrient for plant growth at these locations. The relatively high N:P ratios also support this contention. The soil [P] at all sites included in this study were significantly lower than the global average (Supplementary Data Tables S1 and S2), with most sites exhibiting approximately 10 to 15 % of the global average (He *et al*., 2021). Notably, Kuring-Gai Chase National Park 1, which exhibited the highest soil [P] (accounting for approximately 50 % of the global average, Wright *et al*., 2004), also had elevated concentration of other essential nutrients such as Fe, Mg, Ca, Cu, and Zn. This correlation underscores the complexity of nutrient interactions at different locations and highlights the potential for certain species to adapt to these conditions. Furthermore, all Myrtaceae across the 18 sites displayed high leaf N:P ratios (averaging 28; Fig. 2), much higher than the ratio of 16 or 20 that is broadly considered indicative of P limitation to plant growth (Koerselman and Meuleman, 1996, Güsewell *et al*., 2003, Güsewell, 2004). While our study provides limited information on soil organic matter (SOM) and aluminium (Al) concentrations (Supplementary Data Table S1), we recognise their critical roles in shaping nutrient dynamics in acidic soils of eastern Australia. Elevated Al^3+^ solubility in these acidic soils can induce toxicity, but eucalypts mitigate this risk through exudation of carboxylates (e.g. citrate, malate; Ikka *et al*., 2013), which chelate Al^3+^ into non-phytotoxic complexes while simultaneously enhancing P solubilisation—a dual strategy aligned with Al detoxification mechanisms in plants (Kochian, 1995). SOM further moderates these processes by promoting microbial activity that drives organic P mineralisation and by ectomycorrhizal networks that may enhance nutrient acquisition in Al-rich environments (Soares *et al*., 2024). Future studies integrating comprehensive SOM and Al datasets will clarify edaphic constraints on plant strategies and reinforce the pivotal role of carboxylates in balancing Al tolerance and P mobilisation, key adaptations for dominance in oligotrophic ecosystems.

### Common high leaf [Mn] of *Angophora*, *Eucalyptus* and *Gaudium*

In this study, more than 80 % of all investigated eucalypt species (25 out of 30) exhibited significantly higher leaf [Mn] than the negative reference species, suggesting a pervasive root carboxylate-releasing strategy in forests of south-eastern Australia. Additionally, both *Gaudium* species we sampled and one *Melaleuca* species displayed higher leaf [Mn] than the negative reference species. Further, all *Angophora* species, which are endemic to south-eastern Australia, exhibited significantly higher leaf [Mn] than negative references. Notably, five of the six studied *Angophora* species had leaf [Mn] two to six times greater than that of the positive reference species, clearly indicating strong carboxylate release by roots. Our findings suggest a common occurrence of high leaf [Mn] and consequently a greater root carboxylate-releasing strategy of the *Angophora* species investigated in this study than for other eucalypts. However, it should be noted that our study only included six of all 14 known *Angophora* species (Rejmánek and Richardson, 2019).

For *Eucalyptus*, 17 species had higher leaf [Mn] than the negative reference species, and seven had higher leaf [Mn] than the positive reference. In this study, both *E*. *resinifera* and *E*. *tereticornis* presented high leaf [Mn], and they are known to exude root organic anions under low-P conditions (De Andrade *et al*., 2022). We also found the highest leaf [Mn] for *Eucalyptus* in *E. tereticornis*, which is limited by P in mature forests (Crous *et al*., 2015) and it presents high P-uptake efficiency under low soil P availability (Bulgarelli *et al*., 2019). Five *Eucalyptus* species exhibited no difference of leaf [Mn] compared with negative reference species, and the lowest leaf [Mn] (*E. umbra*) was only 5 % of that in *E. tereticornis* which indicates substantial variation in root carboxylate release activity among *Eucalyptus* species (Bulgarelli *et al*., 2019, Zhou *et al*., 2022, 2024). It is interesting that the species exhibiting the lowest leaf [Mn] occur in habitats that are more biodiverse than the ones with high leaf [Mn] (Rice and Westoby, 1983*a*, *b*). This divergence may also reflect differences in ecological niches and adaptive mechanisms in P-impoverished environments. The weaker correlations observed between leaf metal concentrations in *Eucalyptus* compared with *Angophora* further suggest a distinct physiological strategy in nutrient acquisition.

The observed variation in leaf [Mn] among Myrtaceae in south-eastern Australia may reflect a combination of physiological, edaphic, and ecological factors beyond carboxylate-mediated P mining. While our study highlights carboxylate exudation as a key driver, alternative mechanisms such as genetic variability, complexation with ligands, soil redox conditions and pH dynamics play crucial roles in Mn bioavailability (Gherardi and Rengel, 2004, Schmidt and Husted, 2019, Alejandro *et al*., 2020). Manganese uptake and translocation involve diverse transporter families, including NRAMP, ZIP, and CDF/MTP, which exhibit broad substrate specificity and vary across species (Socha and Guerinot, 2014). Such variability may explain some interspecific differences in Mn accumulation, even among closely related taxa (Nevo and Nelson, 2006). Manganese bioavailability and translocation are also influenced by organic ligands such as nicotianamine, citrate, and malate, which chelate Mn^2+^ and impact its mobility (Fernando *et al*., 2010). In acidic soils, Mn^2+^ becomes more soluble, increasing its uptake by plants (Millaleo *et al*., 2010). However, under oxidising conditions, Mn^2+^ oxidizes to Mn^3+^/Mn^4+^ oxides, reducing its bioavailability (Sutherland *et al*., 2018). At our study sites, acidic soils likely enhanced Mn solubility, but localized redox fluctuations might create microenvironments where Mn availability varies independently of carboxylate exudation (Sparrow and Uren, 2014). For example, *Eucalyptus umbra*'s low leaf [Mn] (63 mg kg^−1^) might reflect root-zone redox dynamics rather than a lack of carboxylate release. Additionally, carboxylates can chelate Mn^2+^ without proton release, decoupling Mn mobilisation from soil acidity (Lambers *et al*., 2015). Given that Australian soils have lower Mn concentrations than temperate forests have (Yan *et al*., 2025), Myrtaceae may exhibit adaptive hyperaccumulation strategies. These alternative explanations suggest a complex interplay of factors influencing Mn dynamics, urging future research to explore soil conditions, microbial communities, and genetic variation in Mn transport to better understand these processes in this biodiverse region.

### Phosphorus-mining strategies through root carboxylate exudation in Myrtaceae

Myrtaceae exhibit a range of nutrient-acquisition strategies, with a significant number of species most likely exhibiting root carboxylate exudation (proxied by leaf [Mn]) to mobilise P, while others potentially rely on neighbouring plants or their mycorrhizal symbionts (Albornoz *et al*., 2017, Gille *et al*., 2024) or possibly disturbance events that raise the soil P availability, e.g. fire (Lambers *et al*., 2022).

Although all sites in this study indicate soil P limitation for plant productivity, the variation in soil conditions across New South Wales (NSW), particularly in terms of soil pH and nutrient availability, significantly influences species distribution (Beadle, 1953, 1962, Le Brocque and Buckney, 1995, Ding *et al*., 2021). The capacity of certain species to exude root carboxylates plays a pivotal role in enhancing P availability in acidic soils. For example, the high [Mn] of *Angophora* species indicates significant carboxylate release, leading to increased P mobilization (Mowatt and Myerscough, 1983). The high leaf [Mn] observed in *Angophora* species, exceeding 1000 mg kg^−1^ in some cases, strongly suggests a reliance on carboxylate-mediated P mining, akin to that of non-mycorrhizal Proteaceae. This finding challenges the long-standing paradigm that mycorrhizal plants (e.g. Myrtaceae) depend solely on fungal symbionts for P acquisition in P-impoverished soils. Instead, *Angophora* appears to employ a dual strategy: while maintaining mycorrhizal associations, it simultaneously releases carboxylates to mine soil P, a trait likely critical for its dominance in highly-weathered acidic soils of south-eastern Australia (Tang *et al*., 2025). This convergence with non-mycorrhizal lineages underscores the evolutionary flexibility of nutrient-acquisition strategies under extreme P limitation (Oliveira *et al*., 2015). The implications extend beyond *Angophora*. Such carboxylate exudation may enable Myrtaceae to exploit microsites where mycorrhizal networks are less effective (e.g. severely P-impoverished habitats). Furthermore, by mobilising P through carboxylates, *Angophora* would alter local soil chemistry, potentially facilitating P uptake for neighbouring plants or influencing competitive dynamics within the ecosystem (Zhou *et al*., 2022). These interactions may explain why *Angophora*-dominated communities often coexist with Proteaceae in fire-prone habitats, despite overlapping nutrient demands. This discovery also raises questions about trade-offs: does carboxylate release incur metabolic costs that constrain growth rates or defence investments in *Angophora*? Comparative studies across Myrtaceae genera (e.g. *Eucalyptus* vs. *Angophora*) could test whether high leaf [Mn] correlates with reduced investment in mycorrhizal partnerships or altered carbon allocation patterns. Resolving these mechanisms will advance predictive models of plant-soil feedback in P-impoverished ecosystems globally (Yan *et al*., 2025).

The strong correlations between leaf [Mn] and [Fe] in *Angophora* (*r* = 0.81, *P* < 0.05) suggest that Fe uptake in *Angophora* is controlled differently from that in other Myrtaceae and most other plants (Hell and Stephan, 2003, Kobayashi and Nishizawa, 2012). Carboxylates like citrate and malate chelate both Fe^3+^ and Mn^2+^, enhancing their solubility and uptake (Lambers *et al*., 2015, Pang *et al*., 2018). Unlike most plants, where Fe and Mn uptake are antagonistic due to competition for transporters (e.g. IRT1 in *Arabidopsis*; Kobayashi and Nishizawa, 2012), *Angophora*'s exudation strategy may synchronize their acquisition. This could reflect adaptive co-regulation to mitigate Fe deficiency in highly weathered soils, where Fe is often immobilized in oxides (Huang *et al*., 2016). The lack of such a correlation in *Eucalyptus* or *Corymbia* highlights *Angophora*'s unique dependency on direct rhizosphere modification. Ecologically, this coordinated Mn-Fe uptake may maintain micronutrient stoichiometry in *Angophora*, supporting metabolic functions like photosynthesis and redox balancing in nutrient-poor habitats (Brandenburg *et al*., 2017). However, it also raises questions about potential trade-offs, such as Mn/Fe toxicity risks in fluctuating soil conditions. These findings underscore the evolutionary innovation of *Angophora* in adapting to Australia's ancient, oligotrophic landscapes and challenge assumptions about universal micronutrient acquisition strategies in mycorrhizal plants. Future work should explore the molecular basis of this synergy (e.g. transporter specificity or carboxylate-metal binding affinity) and its ecological consequences across environmental gradients.

The weak phylogenetic signal for leaf [P] and [Mn] for eucalypts suggests that these traits are evolutionarily labile, similar to the situation in other plant groups such as Australian Proteaceae and Ericaceae (Hayes *et al*., 2021; X.M. Zhou, H. Lambers et al., unpublished data). For example, the species with the lowest leaf [P] were observed in *Eucalyptus* as well as in *Angophora* (*A. hispida*); similarly, we found species with high leaf [Mn] in both genera (*E. tereticornis* and *A. subvelutina*). Interestingly, we found a negative correlation between speciation rate and leaf [P] and leaf [Mn] (Supplementary Data Table S5). This finding suggests that lineages with lower leaf [P], which is indicative of a higher P-use efficiency, have diversified faster in oligotrophic environments. A similar finding was reported for Proteaceae (Hayes *et al*., 2021), suggesting that these adaptations to P-impoverished environments play an important role in the evolution and diversification of Australian plants, and that this is not limited to one particular family. However, these results were not significant for eucalypts once phylogenetic relatedness was taken into account, even though we observed differences across clades. For example, *Eucalyptus* subg. *Eucalyptus* (*E. umbra–E. agglomerata* clade) had the highest speciation rate and generally lowest leaf [P] among the sampled clades of eucalypts (Fig. 5). While our sampling was comprehensive in an eco-physiological context, further sampling of trait data across the phylogeny is required to test these relationships of tip rate and leaf nutrient traits. Expanding sampling for *Blakella* and *Corymbia* should be a priority as we included one of each only in this study. For *Corymbia calophylla* from south-western Australia, Zhou *et al*. (2022) showed a high leaf [Mn] in its natural habitat and carboxylates release in nutrient solution. *Corymbia chippendalei* in the Great Sandy Desert in north-western Australia also has low leaf [P] and high leaf [Mn] compared with other species in that region (Grigg *et al*., 2008). Similarly, targeting additional species in *Gaudium* and close relatives (*Leptospermum sensu stricto.*) would be essential to test whether our findings are applicable to other Myrtaceae lineages. Indeed, another Myrtaceae genus in eastern Australia (*Gossia*) also shows high leaf [Mn] in numerous species (Mclay *et al*., 2018), suggesting this trait may be widespread in the family, but by no means universal (Hayes *et al*., 2014, Shen *et al.*, 2024*a*).

As an ancient and diverse family in Australia, Myrtaceae are commonly associated with ectomycorrhizal (ECM) and/or arbuscular mycorrhizal (AM) fungi (Myerscough, 1998). Some species of *Eucalyptus*, *Corymbia*, *Angophora* and *Melaleuca* are able to associate with both ECM and AM (Brundrett, 2017). It is interesting that the highest leaf [Mn] in this study was found in *G. trinervium*, which is likely AM based on the genus (Moyersoen and Fitter, 1999, Wicaksono *et al*., 2018), but the effect of AM symbiosis on *Gaudium* growth varies across studies, with inconsistent benefits reported (Baylis, 1971, Wicaksono *et al*., 2018). In recent years, there has been a growing interest regarding the role of nutrient uptake from associated fungi in P-limited habitats (Lambers *et al*., 2018, Albornoz *et al*., 2021, Zhou *et al*., 2022, 2024). Despite the significant contribution of mycorrhizal associations to N and water absorption, their ability to acquire P may be restricted in severely P-impoverished environments (Parfitt, 1979, Bolan *et al*., 1984, Treseder and Allen, 2002, Lambers *et al*., 2022), leading to a shift in their primary function towards pathogen defence (Gille *et al*., 2024).

In line with the high leaf [Mn] in some *Eucalyptus* species in Western Australia which were shown to release carboxylates (Lambers *et al*., 2021, Zhou *et al*., 2022), the present results suggest that this P-acquisition strategy is prevalent, not only in eucalypts in south-eastern Australia but also beyond these eucalypt genera and extends to other Myrtaceae such as *Gaudium*. We surmise that the prevalence of root carboxylate-mediated P acquisition far exceeds current claims in the literature, necessitating further investigations encompassing a broader range of species and families, particularly mycorrhizal species and families, and diverse geographical locations, as recently reported for China (Yan *et al*., 2025). Using leaf [Mn] as an initial field screening tool would allow broad surveys, which should be complemented with glasshouse studies (Lambers *et al,* 2021, Zhou *et al*., 2022, Tang *et al*., 2025, Wang *et al*., 2025).

## Supplementary Material

mcaf129_Supplementary_Data

## Data Availability

The data that support the findings of this study are available in Supplementary Information.

## SUPPLEMENTARY DATA

Supplementary data are available at *Annals of Botany* online and consist of the following. Table S1: Bulk soil characteristics from 18 sites. Table S2: Published soil and leaf phosphorus (P) concentrations related to this study and the references. Table S3: Multiple models were employed in this study to calculate the Akaike's Information Criterion corrected for finite sample sizes (AICc). Table S4: Summary statistics for phylogenetic signal tests (Blomberg's K) of leaf phosphorus concentrations ([P]) and manganese concentrations ([Mn]) for eucalypts. Table S5: Summary statistics for Spearman rank correlation tests between speciation tip rates and leaf nutrient traits. Table S6: Summary statistics for phylogenetic generalised least square regression (PGLS) regressions accounting for phylogenetic relatedness. Figure S1: Site-average leaf nitrogen (N, A) and phosphorus (P, B) concentrations as well as the leaf N:P ratio (C). Figure S2: Species-level leaf nitrogen (N, A) and phosphorus (P, B) concentrations as well as the leaf N:P ratio (C) for different Myrtaceae species across 18 sites from three regions. Figure S3: Leaf manganese (Mn) concentration of targeted Myrtaceae relative to each site-specific negative reference where the value of a target species was subtracted from the mean of the negative reference. Figure S4: Leaf manganese (Mn) concentration of targeted Myrtaceae relative to each site-specific positive reference where the value of a target species was subtracted from the mean of the positive reference. Figure S5: Relative leaf manganese (Mn) concentration ([Mn]) of targeted Myrtaceae. Leaf [Mn] of targeted species across sites was standardised to the absolute leaf [Mn] using the leaf [Mn] of respective positive and negative references at each site using Equation 1. Figure S6: Cross-correlations of leaf nutrient concentrations and soil parameters. Figure S7: Mixed effects models of genus (a) and regions (b) on leaf manganese (Mn) concentration ([Mn]). Figure S8. Maximum likelihood ancestral reconstructions of speciation tip rates derived from BAMM for eucalypts.
