# Supplementary material for: Leaf manganese concentrations reveal phosphorus-mining strategies and trait diversification of Myrtaceae in south-eastern Australia
Source: Ann Bot. 2025 Jun 17;136(5-6):1367–81. doi: 10.1093/aob/mcaf129 (PMC12682836; doi:10.1093/aob/mcaf129)
Supplement: mcaf129_Supplementary_Data [file mcaf129_supplementary_data.zip › Yan et al_Supplementary Information.docx]

**Supplementary Information**

**Leaf manganese concentrations reveal** **phosphorus-mining strategies and trait diversification of Myrtaceae in south-eastern Australia**

**Supplemental Table S1**. Bulk soil characteristics from 18 sites

| Site | Region | Site name | pH (H_2_O) | pH (CaCl_2_) | EC  dS m^-1^ | Total P  mg kg^-1^ | Olsen P  mg kg^-1^ | Mn  mg kg^-1^ | Fe  mg g^-1^ | Mg  mg kg^-1^ | Ca  mg kg^-1^ | K  mg kg^-1^ | Cu  mg kg^-1^ | Zn  mg kg^-1^ |
| --- | --- | --- | --- | --- | --- | --- | --- | --- | --- | --- | --- | --- | --- | --- |
| 1 | Western Sydney | EucFACE | 5.0(0.1) | 4.6(0.1) | 0.03(0.003) | 64(7) | 2.3(0.2) | 24.7(7.3) | 2.2(0.4) | 90(11) | 74(14) | 137(9) | 2.4(0.1) | 3.8(0.4) |
| 2 | Blue Mountains | Davies Park | 5.1(0.1) | 4.5(0.1) | 0.02(0.002) | <50 | 1.2(0.1) | 4.4(0.4) | 2.6(0.3) | 94(5) | 57(4) | 133(14) | 1.1(0.1) | 2.3(0.3) |
| 3 | Blue Mountains | Grose Road | 5.0(0.1) | 4.5(0.1) | 0.03(0.001) | <50 | 1.4(0.2) | 4.6(0.3) | 7.3(0.5) | 131(11) | 68(11) | 205(13) | <1.0 | 3.1(0.4) |
| 4 | Blue Mountains | Minnehaha1 | 4.7(0.1) | 4.3(0.1) | 0.03(0.002) | 60(7) | 1.5(0.1) | 5.0(0.5) | 7.6(0.9) | 124(11) | 51(1) | 114(10) | 2.0(0.2) | 2.9(0.2) |
| 5 | Blue Mountains | Minnehaha2 | 4.9(0.1) | 4.5(0.1) | 0.03(0.003) | <50 | 2.0(0.2) | 12.1(0.7) | 15.6 (0.9) | 210(27) | 59(5) | 140(10) | 1.3(0.1) | 4.8(0.2) |
| 6 | Western Sydney | Castlereagh Reserve1 | 5.3(0.1) | 4.7(0.1) | 0.02(0.001) | <50 | 1.2(0.1) | 10.4(1.4) | 18.6(3.3) | 112(6) | 51(2) | 57(3) | 1.6(0.2) | 3.0(0.2) |
| 7 | Western Sydney | Castlereagh Reserve2 | 5.3(0.1) | 4.5(0.1) | 0.03(0.001) | 62(6) | 1.8(0.2) | 19.1(1.4) | 35.8(8.3) | 297(32) | 74(6) | 168(14) | 3.5(0.5) | 6.0(0.4) |
| 8 | Coastal | Kuringai Chase NP1 | 5.6(0.2) | 4.9(0.2) | 0.06(0.006) | 313(77) | 2.9(0.2) | 109.4(29.9) | 65.3(16.1) | 781(164) | 1357(428) | 204(30) | 6.5(1.9) | 14.2(3.4) |
| 9 | Coastal | Kuringai Chase NP2 | 4.6(0.1) | 4.0(0.1) | 0.04(0.003) | <50 | 1.5(0.2) | 2.2(0.5) | 2.1(0.7) | 87(13) | 56(4) | 273(33) | <1.0 | 2.4(0.4) |
| 10 | Blue Mountains | Euroka1 | 5.4(0.1) | 4.6(0.1) | 0.03(0.003) | 63(5) | 1.9(0.1) | 19.2(2.6) | 8.3(1.1) | 217(33) | 128(29) | 189(18) | 1.5(0.1) | 4.9(0.2) |
| 11 | Blue Mountains | Euroka2 | 5.0(0.2) | 4.3(0.2) | 0.02(0.003) | <50 | 1.7(0.1) | 7.0(0.9) | 2.6(0.5) | 97(9) | 66(7) | 136(11) | 1.4(0.1) | 4.5(0.3) |
| 12 | Blue Mountains | Lapstone | 5.0(0.1) | 4.2(0.1) | 0.03(0.004) | 94(10) | 2.5(0.1) | 18.8(6.0) | 6.4(1.3) | 238(39) | 186(63) | 280(32) | 4.0(0.7) | 8.7(1.3) |
| 13 | Coastal | Dog Pound Creek Reserve1 | 5.2(0.1) | 4.3(0.1) | 0.04(0.003) | <50 | 1.6(0.1) | 6.5(1.0) | 3.2(0.2) | 159(20) | 508(165) | 115(9) | 1.9(0.2) | 7.5(0.9) |
| 14 | Coastal | Dog Pound Creek Reserve2 | 4.2(0.1) | 3.4(0.1) | 0.07(0.005) | 70(6) | 9.1(1.9) | 2.2(0.1) | 1.0(0.2) | 98(15) | 98(22) | 82(8) | 1.0(0.1) | 4.8(1.3) |
| 15 | Coastal | Kuringai Wildflower Garden1 | 4.6(0.1) | 4.0(0.1) | 0.04(0.003) | <50 | 1.5(0.3) | 5.2(2.2) | 2.1(1.0) | 78(9) | 56(7) | 147(16) | 1.5(0.2) | 3.9(0.7) |
| 16 | Coastal | Kuringai Wildflower Garden2 | 4.7(0.2) | 3.9(0.2) | 0.03(0.005) | <50 | 1.2(0.1) | 4.1(1.3) | 2.7(1.1) | 91(19) | 77(21) | 104(15) | 1.0(0.1) | 2.9(0.9) |
| 17 | Coastal | Kuringai Wildflower Garden3 | 5.4(0.1) | 4.7(0.1) | 0.05(0.008) | 53(2) | 1.9(0.1) | 12.1(0.6) | 9.7(0.3) | 367(32) | 467(57) | 238(10) | 2.1(0.3) | 9.1(0.8) |
| 18 | Coastal | Kuringai Wildflower Garden4 | 5.1(0.1) | 4.4(0.1) | 0.04(0.002) | 55(4) | 1.5(0.1) | 5.9(1.1) | 7.2(2.5) | 164(12) | 211(56) | 181(14) | 1.6(0.3) | 6.6(0.6) |

N= 5 for each site; values in brackets are SE. EC, Electrical Conductivity; P, phosphorus; Mn, manganese; Fe, iron; Mg, magnesium; Ca, calcium; K, potassium; Cu, copper; Zn, zinc. Al, aluminum; OM, organic matter.

For Kuringgai Chase NP1: Al (62 mg g^-1^), OM (142 mg g^-1^); Kuringgai Chase NP2: Al (8.9 mg g^-1^), OM (33 mg g^-1^).

**Supplemental Table S2**. Published soil and leaf phosphorus (P) concentrations related to this study and the references.

| Site | Soil type | Soil total P  mg kg^-1^ | Plant-available soil P mg kg^-1^ | Leaf P  mg g^-1^ |
| --- | --- | --- | --- | --- |
| Global average | - | 570 (He *et al.*, 2021) | - | - |
| EucFACE | Alluvia | - | 9.3 (Batjes, 2011) | 0.7 (Jiang *et al.*, 2024) |
| Kuring-gai Chase NP | Sandstone | 94 (Wright *et al.*, 2001) | 1.2 (Tsujii *et al.*, 2024) | 0.2 (Tsujii *et al.*, 2024) |
| Kuring-gai Chase NP | Basalt | 442 (Wright *et al.*, 2001) | 3.4 (Tsujii *et al.*, 2024) | 0.4 (Tsujii *et al.*, 2024) |
| Blue Mountains | Sandstone | - | - | 0.5 (*Acacia suaveolens*), 0.2 (*Banksia serrata*) (de Campos *et al.*, 2013) |
| Blue Mountains | Sandstone | 84 (Dhakal *et al.*, 2025) | 2.3 (Dhakal *et al.*, 2025) | 0.2 – 0.6 mg g^-1^ (Dhakal *et al.*, 2025) |

**Supplemental Table S3.** Multiple models were employed in this study to calculate the Akaike’s Information Criterion corrected for finite sample sizes (AICc). The R codes provided below demonstrate the calculation using leaf Mn concentration as an example.

| **Models** | **Codes** |
| --- | --- |
| Model_1 | gls (leafMn ~ Species, data =MyrtaceaePro, na.action=na.omit) |
| Model_2 | update (mod.leafmn.1, weights = varIdent(form = ~ 1 \| Species)) |
| Model_3 | update (mod.leafmn.1, weights = varPower()) |
| Model_4 | update (mod.leafmn.1, weights = varConstPower()) |
| Model_5 | update (mod.leafmn.1, weights = varPower(form = ~ fitted(.) \| Species)) |
| Model_6 | update (mod.leafmn.1, weights = varComb(varIdent(form = ~ 1 \| Species), varPower())) |
| Model_7 | update (mod.leafmn.1, weights = varExp()) |
| Model_8 | update (mod.leafmn.1, weights = varExp(form = ~ fitted(.) \| Species)) |

**Supplemental Table S4.** Summary statistics for phylogenetic signal tests (Blomberg’s K) of leaf phosphorus concentrations ([P]) and manganese concentrations ([Mn]) for eucalypts. Statistically significant p-values are indicated in bold and ‘*’.

|  | leaf.P (log) | | leaf.Mn (log) | |
| --- | --- | --- | --- | --- |
| test | phylo signal | p-value | phylo signal | p-value |
| Blomberg's K (nsim = 10000) | 0.203 | 0.135 | 0.2418 | **0.027*** |

**Supplemental Table S5.** Summary statistics for Spearman rank correlation tests between speciation tip rates and leaf nutrient traits. Statistically significant p-values are indicated in bold and ‘*’.

| **Spearman** | **rho** | **p-value** |
| --- | --- | --- |
| log.tiprates~log.P | -0.392 | **0.044*** |
| log.tiprates~log.Mn | -0.259 | 0.191 |

**Supplemental Table S6.** Summary statistics for phylogenetic generalised least square regression (PGLS) regressions accounting for phylogenetic relatedness.

| **PGLS** | **AIC** | **LogLik** | **Std. Error** | **t-value** | **estimate** | **p-value** |
| --- | --- | --- | --- | --- | --- | --- |
| tip rates explained by leaf [P] | -168.25 | 87.12 | 0.007 | -0.19 | -0.00144 | 0.848 |
| tip rates explained by leaf [Mn] | -166.93 | 86.46 | 0.00389 | -0.134 | -0.000052 | 0.8943 |


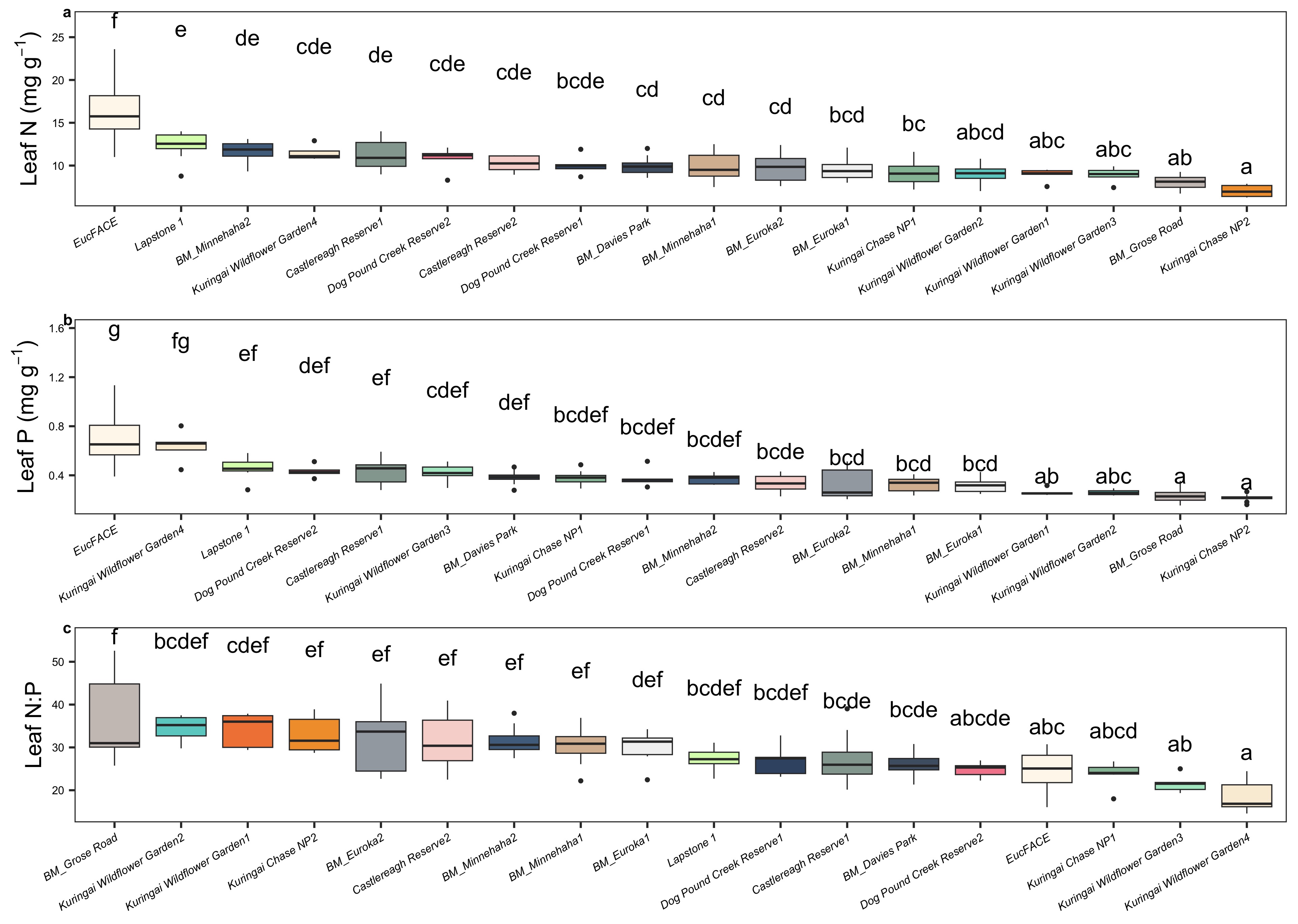


**Figure S1.** Site-average leaf nitrogen (N, a) and phosphorus (P, b) concentrations as well as the leaf N:P ratio (c) across 18 sites. The analysis excluded reference species; n= 193 individuals across 34 different Myrtaceae species. Different letters indicate significant differences among sites (*p*<0.05).





**Figure S2.** Leaf nitrogen (N, a) and phosphorus (P, b) concentrations as well as the leaf N:P ratio (c) for different species across 18 sites. The analysis excluded reference species; n= 193 individuals across 34 different Myrtaceae species.


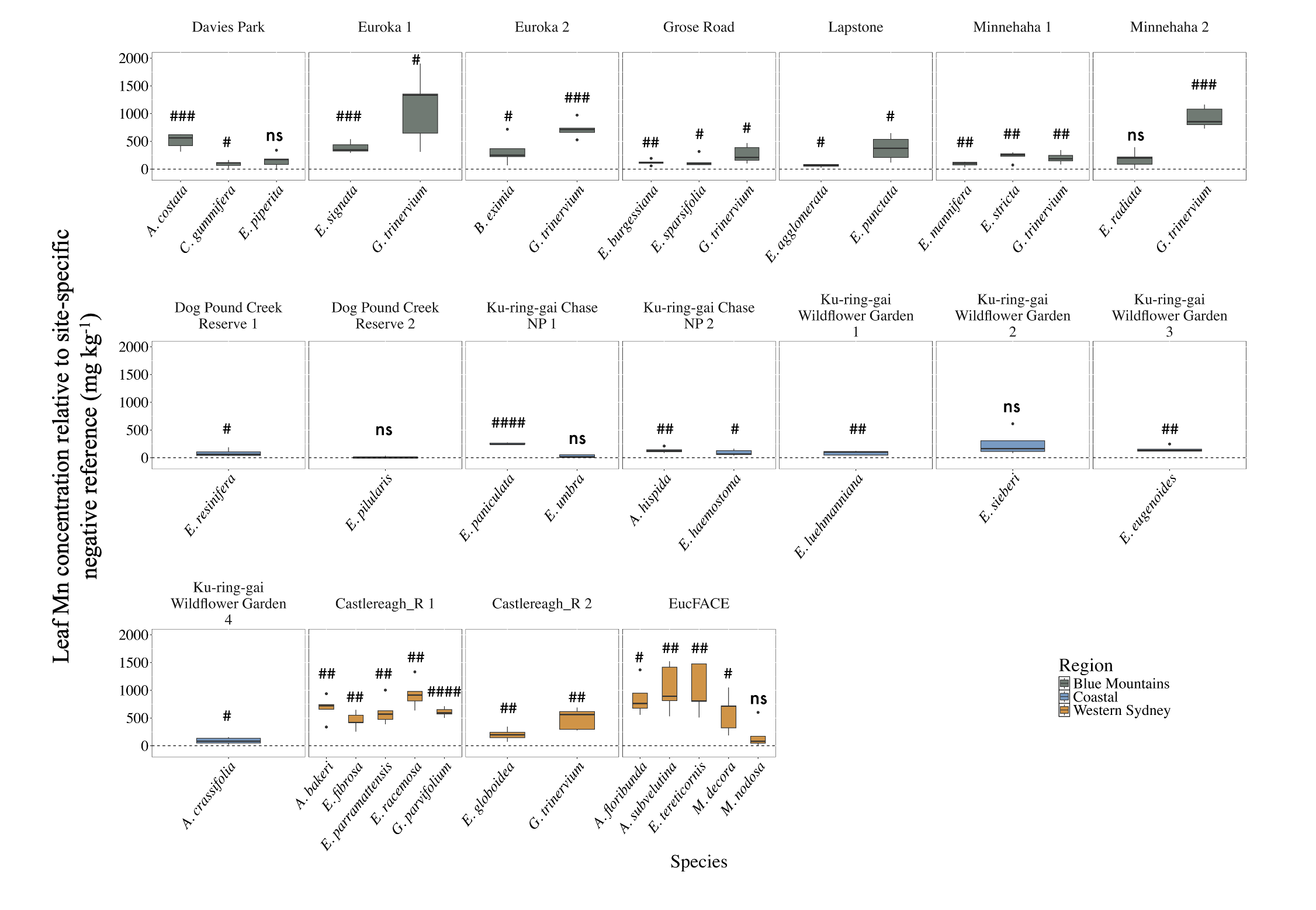


**Figure S3.** Leaf manganese (Mn) concentration of targeted Myrtaceae relative to each site-specific **negative** reference where the value of a target species was subtracted from the mean of the negative reference. Values of zero in the figure indicate parity with the mean [Mn] of the negative reference, hence indicating no carboxylate exudation. Specific information of references at each site can be found in Fig. S2. The Welch t-test was used to compare the significance between target species and positive references (#, *p*<0.05; ##, *p*<0.01; ### *p*<0.001; ####, *p*<0.0001) at the same site in the field; ns indicates no significant difference


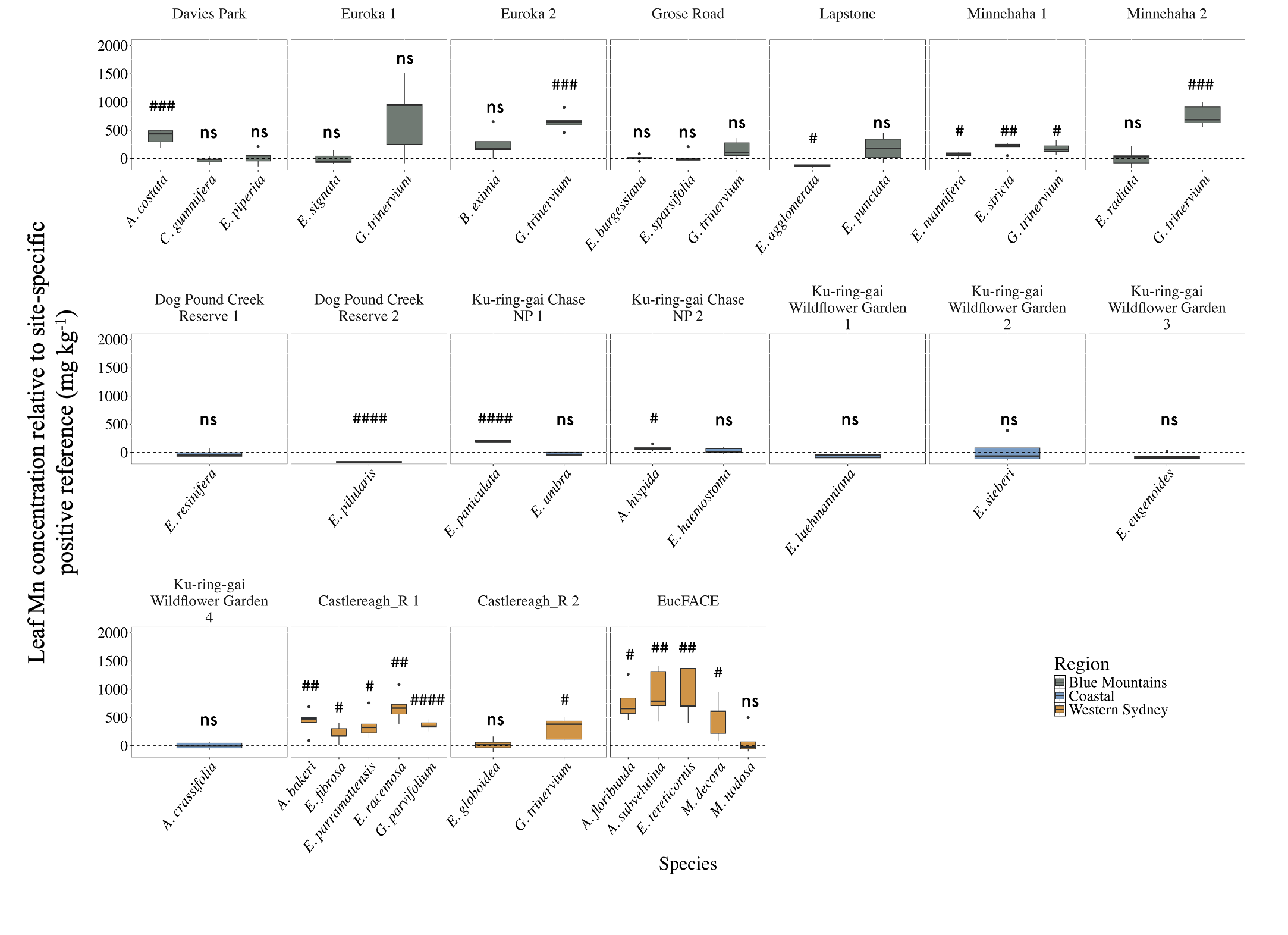


**Figure S4.** Leaf manganese (Mn) concentration of targeted Myrtaceae relative to each site-specific **positive** reference where the value of a target species was subtracted from the mean of the positive reference. Values significantly higher than zero indicate a strong capacity to exude root carboxylates for the species. Specific information of references at each site can be found in Fig. S2. The Welch t-test was used to compare the significance between target species and positive references (#, *p*<0.05; ##, *p*<0.01; ### *p*<0.001; ####, *p*<0.0001) at the same site in the field; ns indicates no significant difference





**Figure S5**. Relative leaf manganese concentration ([Mn]) of targeted Myrtaceae. Leaf [Mn] values were standardized using Equation 1:

$$Relative leaf \left[ \mathrm{Mn} \right]\left( \% \right)=\frac{\mathrm{Target}\left[ \mathrm{Mn} \right]- Negative reference \left[ \mathrm{Mn} \right]}{Positive reference \left[ \mathrm{Mn} \right]- Negative reference \left[ \mathrm{Mn} \right]}\times100$$

Where **Target [Mn]** is the manganese concentration of the focal species, **Negative reference [Mn]** is the mean [Mn] of non-carboxylate-exuding reference species (e.g., *Xanthorrhoea*), and **Positive reference [Mn]** is the mean [Mn] of carboxylate-exuding species (e.g., *Banksia*).


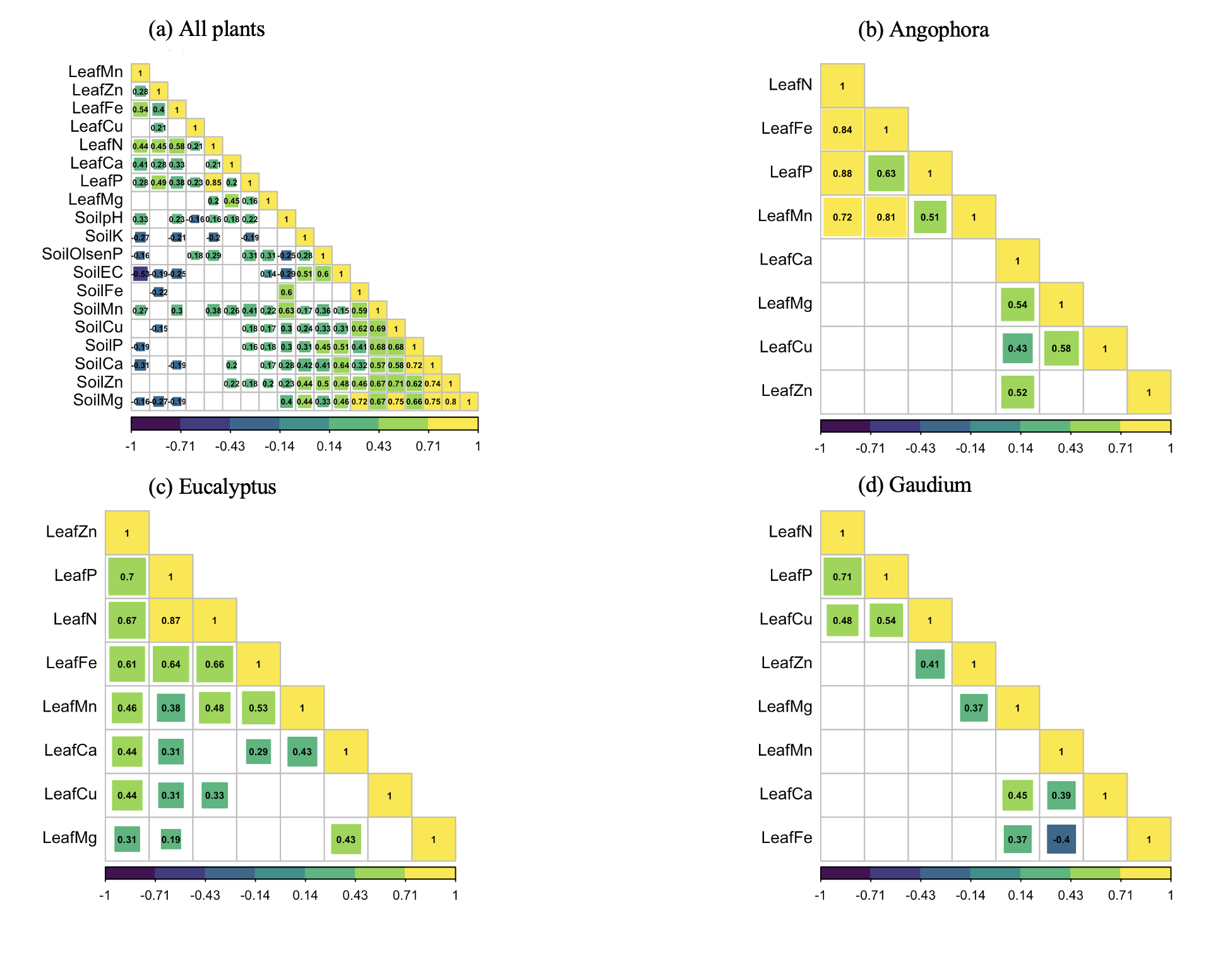


**Figure S6.** Cross-correlations of leaf nutrient concentrations and soil parameters, log transformed; (a), n=193 for plant samples, and n=90 for soil samples; (b), n=29 for *Angophora*; (c), n=109 for *Eucalyptus*; (d), n=35 for *Gaudium*. *p*<0.05.


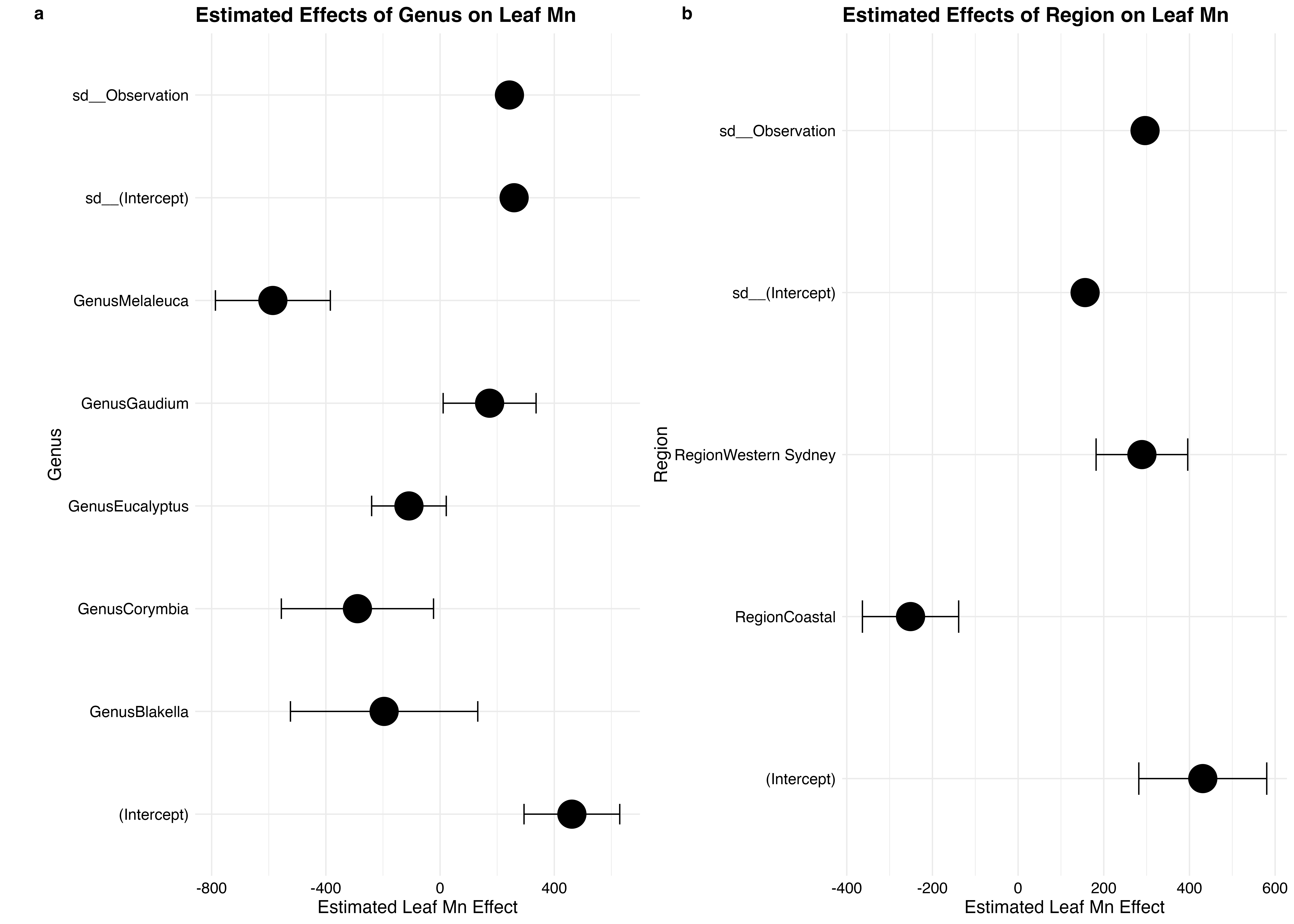


**Figure S7.** Mixed effects models of genus (a) and regions (b) on leaf manganese (Mn) concentration ([Mn]). All genera had a significant effect on leaf [Mn] (*p*<0.05), except *Eucalyptus* and *Blakella*; Western Sydney and Coastal presented significant effect on leaf [Mn] (*p*<0.05).

**Figure S8.** Maximum likelihood ancestral reconstructions of speciation tip rates derived from BAMM for eucalypts, performed using the *contMap* function in phytools (Revell 2012b). We used the published phylogeny of Thornhill et al. (2019).

**References**

**Batjes N. 2011.** Overview of soil phosphorus data from a large international soil database: ISRIC-World Soil Information.

**Dhakal, S., Mehnaz K.R., Rogers E.I.E., Wright I.J., Ellsworth, D.S**. Efficient or proficient? Leaf phosphorus recycling among species in a nutrient-poor, species-rich community. *Functional Ecology* (submitted).

**de Campos MCR, Pearse SJ, Oliveira RS, Lambers H. 2013.** Downregulation of net phosphorus-uptake capacity is inversely related to leaf phosphorus-resorption proficiency in four species from a phosphorus-impoverished environment. *Annals of Botany* **111**(3): 445-454.

**He XJ, Augusto L, Goll DS, Ringeval B, Wang YP, Helfenstein J, Huang YY, Yu KL, Wang ZQ, Yang YC, et al. 2021.** Global patterns and drivers of soil total phosphorus concentration. *Earth System Science Data* **13**(12): 5831-5846.

**Jiang M, Crous KY, Carrillo Y, Macdonald CA, Anderson IC, Boer MM, Farrell M, Gherlenda AN, Castaneda-Gomez L, Hasegawa S, et al. 2024.** Microbial competition for phosphorus limits the CO_2_ response of a mature forest. *Nature* **630**(8017): 660-665.

**Thornhill AH, Crisp MD, Külheim C, Lam KE, Nelson LA, Yeates DK, Miller JT**.**2019** A dated molecular perspective of eucalypt taxonomy, evolution and diversification. Aust Syst Bot 32 (1):29-48. doi:10.1071/Sb18015

**Tsujii Y, Atwell BJ, Lambers H, Wright IJ. 2024.** Leaf phosphorus fractions vary with leaf economic traits among 35 Australian woody species. *New Phytologist* **241**(5): 1985-1997.

**Wright IJ, Reich PB, Westoby M. 2001.** Strategy shifts in leaf physiology, structure and nutrient content between species of high- and low-rainfall and high- and low-nutrient habitats. *Functional Ecology* **15**(4): 423-434.
